# Retrospective cohort study of the efficacy and safety of dabigatran: real-life dabigatran use including very low-dose 75 mg twice daily administration

**DOI:** 10.1186/s40780-019-0145-3

**Published:** 2019-08-01

**Authors:** Yuuki Akagi, Tatsuo Chiba, Shusuke Uekusa, Hiroyoshi Kato, Shigeo Yamamura, Yukiko Aoki, Mizuho Enoki, Yuka Ogawara, Takanori Kasahara, Yuki Kimura, Tadahiro Shimizu, Aiko Takeishi, Yuko Nakajima, Hideki Kobayashi, Kaoru Sugi

**Affiliations:** 1 Department of Pharmacy, National Hospital Organization, Yokohama Medical Center, 3-60-2 Harajuku, Totsuka, Yokohama, Kanagawa 245-8575 Japan; 2 Department of Pharmacy, Toho University Ohashi Medical Center, 2-17-6 Ohashi, Meguro, Tokyo, 153-8515 Japan; 3 Faculty of Pharmaceutical Sciences, Toho University, 2-2-1 Miyama, Funabashi, Chiba, 274-8510 Japan; 4 Faculty of Pharmaceutical Sciences, Josai International University, 1 Gumyo, Togane, Chiba, 283-8555 Japan; 5 Department of Pharmacy, National Hospital Organization, Tokyo Medical Center, 2-5-1 Higashigaoka, Meguro, Tokyo, 152-8902 Japan; 6 Department of Pharmacy, Tamagawa Hospital, 4-8-1 Seta, Setagaya, Tokyo, 158-0095 Japan; 7 Department of Pharmacy, Mishuku Hospital, 5-33-12 Kami-meguro, Meguro, Tokyo, 153-0051 Japan; 8 Department of Pharmacy, Kohsei Chuo General Hospital, 1-11-7 Mita, Meguro, Tokyo, 153-8581 Japan; 9 Department of Cardiovascular Medicine, Odawara Cardiovascular Hospital, 6-1-14 Yahagi, Odawara, Kanagawa 250-0873 Japan

**Keywords:** Dabigatran, Anticoagulant, Excessive dose reduction, Thromboembolism, Non-valvular atrial fibrillation, Retrospective study

## Abstract

**Background:**

Dabigatran is a direct thrombin inhibitor and an anticoagulant that is prescribed to prevent ischemic stroke and systemic embolism in non-valvular atrial fibrillation. Dabigatran (150 mg twice daily) is non-inferior to warfarin for the prevention of stroke and systemic embolism. A dose reduction to 110 mg twice daily should be considered for patients with decreased renal function, elderly patients, and those with a history of gastrointestinal bleeding. A small number of patients are prescribed 75 mg twice daily; however, excessive dose reduction below that indicated on the package insert may decrease the effectiveness of dabigatran. In this study, we investigated the incidence of thromboembolic events and hemorrhagic complications in patients receiving different doses of dabigatran, including patients receiving the very low-dose of 75 mg twice daily.

**Methods:**

Five hospitals in Meguro and Setagaya areas of Tokyo were included in this study. The subjects were patients receiving dabigatran in the hospitals from March 2011 to February 2014. Thromboembolic events (stroke, systemic embolism, and transient cerebral ischemic attack) and hemorrhagic complications occurring before December 2014 were retrospectively evaluated.

**Results:**

A total of 701 subjects received dabigatran during the study period: 187 patients (26.7%) received 150 mg twice daily (normal dose), 488 patients (69.6%) received 110 mg twice daily (low-dose), and 26 patients (3.7%) received 75 mg twice daily (very low-dose). Thromboembolism occurred in 4 (2.1%), 11 (2.3%), and 3 patients (11.5%), in the normal dose, low-dose, and very low-dose groups, respectively. The odds ratio of the 75 mg dose to the 150 and 110 mg doses was 5.73 (95% CI, 1.55–21.2; *p = 0.009*), and the incidence with the 75 mg dose was higher than that with the other doses. Although the number of events was limited, it should be noted that 3 patients in the very low-dose group had thromboembolic events.

**Conclusions:**

The results suggest that sufficient anticoagulation efficacy may not be maintained when the dabigatran dose is excessively reduced to 75 mg twice daily.

## Background

Atrial fibrillation (AF) is an arrhythmia commonly reported in the elderly. Cardiogenic ischemic stroke is often severe because it is characterized by larger lesions than other types of stroke, and its recurrence rate is higher than that of other stroke types [1]. Anticoagulant therapy with warfarin can reduce the risk of stroke by more than 60% in patients with moderate to high AF risk [2]. However, since the anticoagulant effect of warfarin is greatly affected by diet and many drugs [3], it fluctuates easily. In fact, consuming food and drinks containing vitamin K such as natto (fermented soybeans), chlorella, and aojiru (green juice), may reduce the anticoagulant effect, whereas the use of concomitant medications, such as antibiotics and CYP2C9 inhibitors, may enhance anticoagulant effect. In addition, the therapeutic range of warfarin is narrow; therefore, poor anticoagulation control is common. Because of these limitations, new direct oral anticoagulants (DOACs) that directly inhibit a single coagulation factor have been recently discovered. The DOACs dabigatran, rivaroxaban, apixaban, and edoxaban are currently available in Japan.

Dabigatran (Prazaxa®) is a direct thrombin inhibitor, which has been marketed in Japan since 2011. It is prescribed to prevent ischemic stroke and systemic embolism in patients with non-valvular AF. In the Randomized Evaluation of Long-term Anticoagulant Therapy (RE-LY) clinical trial, dabigatran (150 mg twice daily) was shown to be non-inferior to warfarin for the prevention of stroke and systemic embolism [4]. A subgroup analysis of Asian patients in the RE-LY trial showed that the rate of stroke or systemic embolism with dabigatran (150 mg twice daily) was lower than that with warfarin [5]. For patients with risk factors such as decreased renal function, advanced age, and history of gastrointestinal bleeding, the risk of hemorrhagic complications with dabigatran is higher; therefore, a dose reduction to 110 mg twice daily is recommended on the package insert of Prazaxa® [6, 7].

However, very few patients are prescribed dabigatran at a very low dose of 75 mg twice daily, probably due to safety concerns. One of the reasons is that dabigatran is available on the market in Japan as a 75 mg capsule, not as a 150 mg capsule. The patients in the RE-LY trial were assigned to receive 150 mg or 110 mg twice-daily dabigatran or warfarin treatment regardless of renal function [4]. However, current clinical doses for AF in the United States of America depend on renal function: 150 mg of dabigatran twice daily for creatinine clearance over 30 mL/min and 75 mg twice daily for creatinine clearance of 15 to 30 mL/min [8]. The dose of 75 mg twice daily occasionally prescribed in Japan may be based on the American dose recommendation. Because 75 mg twice daily is not an approved dose of dabigatran in Japan and its efficacy and safety are unknown, the anticoagulant effect of dabigatran may decrease with the lower dose. In this study, we investigated the incidence of thromboembolic events and hemorrhagic complications with dabigatran, including the use of the 75 mg twice daily dose.

## Methods

### Data sources

Five differently sized hospitals in the Meguro or Setagaya areas of Tokyo were enrolled in this study (Toho University Ohashi Medical Center, National Hospital Organization Tokyo Medical Center, Tamagawa Hospital, Mishuku Hospital, and Kohsei Chuo General Hospital). This multicenter, retrospective cohort study was performed after approval from each hospital’s institutional review board (approval no. 15–46, Toho University Ohashi Medical Center; the representative hospital in our research team). Data were analyzed at the National Hospital Organization Yokohama Medical Center.

### Inclusion and exclusion criteria

The subjects started dabigatran in any of the 5 hospitals between March 2011 and February 2014. We excluded patients who had a dabigatran prescription history of less than 1 month, were hospitalized except for cardiovascular and cerebrovascular diseases, and had no information available after hospital discharge. Subjects with a description of poor medication adherence (less than 80%) in their medical records were also excluded.

### Data collection

The following data were collected: age, sex, body weight, serum creatinine, activated partial thromboplastin time (aPTT), starting dose of dabigatran, date (year and month) of dabigatran initiation, the therapy period of dabigatran, any previous anticoagulant therapy before starting dabigatran, history of bleeding, incidence of complications (heart failure, hypertension, diabetes, and cerebral vascular disease for CHADS_2_ scores [9]), concomitant medications [antiplatelet drugs (low-dose aspirin, ticlopidine, and clopidogrel) and P-glycoprotein inhibitors (verapamil, amiodarone, cyclosporine, tacrolimus, itraconazole, and clarithromycin)], and incidence of thromboembolic and bleeding events. Creatinine clearance (CCr) was calculated using the Cockcroft-Gault formula. Patients were classified into 3 groups: normal dabigatran dose (300 mg/day; 150 mg twice daily), low-dose dabigatran (220 mg/day; 110 mg twice daily), and very low-dose dabigatran (150 mg/day; 75 mg twice daily). The incidence of thromboembolic events included stroke, transient ischemic attacks (TIA), and systemic embolism as defined by the RE-LY trial. In addition, the patients were monitored for major and minor bleeding. Major bleeding was defined as a decrease in hemoglobin concentration ≥ 2.0 g/dL, transfusion of whole or concentrated blood, and symptomatic hemorrhage of a vital organ or intracranial hemorrhage [4], whereas minor bleeding was defined as other hemorrhagic events.

### Primary and secondary outcomes

The primary outcomes were the incidence of thromboembolic events during the period between dabigatran initiation and December 2014. Side effects of bleeding were also investigated. Risk of developing cerebral infarction among the groups was adjusted by CHADS_2_ scores. As a secondary outcome, the effect of patient background on the incidence of thromboembolic events was also investigated.

### Statistical analysis

T-test (quantitative variable), Fisher’s exact test (qualitative variable, values in any of cells are 10 or below), or chi-square test (qualitative variable, others) was used to estimate clinical characteristics of patients. We compared the risk between normal and low-dose dabigatran (150 mg and 110 mg twice daily) and very low-dose dabigatran (75 mg twice daily) because the incidence of thromboembolic events in the 75 mg twice daily group was assumed to be higher than that in the other groups. Multivariable logistic regression analysis was performed to estimate the thromboembolic and bleeding risk of excessive dose reduction of dabigatran, using variables which are selected by stepwise logistic regression method. Statistical analysis was carried out using JMP Pro 14.2.0 (SAS institute Japan Ltd), and *p* values below 5% were considered to be significant.

## Results

### Patients’ characteristics

One hundred and eighty seven patients (26.7%) received 150 mg dabigatran twice daily (normal dose group), and 488 patients (69.6%) received 110 mg twice daily (low-dose group). Only a few patients (26 patients, 3.7%) received 75 mg twice daily (very low-dose group). Clinical characteristics of patients in this study are shown in Table 1, and 150 and 110 mg twice daily groups are shown as one group as mentioned above. The average age of 150 and 110 mg twice daily group (normal dose and low-dose group) was 70.8 ± 10.8 years (mean ± S.D.); the lower dose was associated with a higher average age. The CCr of 150 and 110 mg twice daily group was 69.4 ± 25.3 mL/min; CCr declined in the lower dose groups.

**Table 1:** Clinical characteristics of patients

Variables	Total (*n* = 701)	150 mg and 110 mg twice daily (*n* = 675)	75 mg twice daily (*n* = 26)	*P*-value
Age, years	71.1 ± 10.9	70.8 ± 10.8	78.6 ± 10.0	0.004^**^
Gender, female (%)	244 (34.8%)	231 (34.2%)	13 (50.0%)	0.098^*^
Creatinine clearance: Ccr (mL/min)	68.7 ± 25.3	69.4 ± 25.3	53.5 ± 20.9	0.003^*^
History of bleeding	79 (11.3%)	74 (11.0%)	5 (19.2%)	0.191^***^
New users	354 (50.5%)	348 (51.6%)	6 (23.1%)	0.004^***^
Change from warfarin	232 (33.1%)	222 (32.9%)	10 (38.5%)	0.532^***^
C: Congestive heart failure	138 (19.7%)	128 (19.0%)	10 (38.5%)	0.002^***^
H: Hypertension	426 (60.8%)	406 (60.1%)	20 (76.9%)	0.041^**^
A: Aged 75 or older	294 (41.9%)	278 (41.2%)	16 (61.5%)	0.021^**^
D: Diabetes Mellitus	140 (20.0%)	133 (19.7%)	7 (26.9%)	0.117^***^
S: Stroke/TIA (2 points)	185 (26.4%)	177 (26.2%)	8 (30.8%)	0.327^***^
CHADS2 score	1.95 ± 1.35	1.92 ± 1.33	2.54 ± 1.77	0.011^*^
With antiplatelet agents	129 (18.4%)	125 (18.5%)	4 (15.4%)	0.686^***^

Mean ± S.D., ^*^ t-test, ^**^ Chi-square test, ^***^ Fisher’s exact test

Overall, half of the patients were new users of anticoagulant therapy, and one-third switched to dabigatran from warfarin. Warfarin and dabigatran were the only oral anticoagulants available in the first half of the study period. Other anticoagulants were available in the second half, but other DOACs are not prescribed much due to their recent availability in Japan.

### Thromboembolic events

The incidence of thromboembolic events during this study period is shown in Table 2. Thromboembolism occurred in 4 patients (2.1%) in the normal dose group and in 11 patients (2.3%) in the low-dose group (15 patients in the normal and low-dose group). On the other hand, 3 of 26 patients (11.5%) in the very low-dose group had a thromboembolic event. The odds ratio of the 75 mg group to the 150 and 110 mg twice daily groups was 5.73 [95% confidence interval (CI), 1.55–21.2; *p = 0.009*]; however, the number of events was limited. The CHADS_2_ score in 150 and 110 mg twice daily group was 1.92 ± 1.33 (Table 1); the lower dose was associated with a higher CHADS_2_ score. The unit odds ratio of the CHADS_2_ score was 1.69 [95% CI, 1.21–2.37; *p = 0.002*].

**Table 2:** Thromboembolic events by dabigatran dose and each factor

Factor	Thromboembolism (+) (*n* = 18)	Thromboembolism (−) (*n* = 683)	ODDS ratio [95% CI], (*P*-value)
Very low-dose (75 mg twice daily)	3	23	5.73 [1.55–21.2], (0.009*)
Age, years	77.4 ± 13.5	71.0 ± 10.8	1.06 [1.01–1.12], (0.013*)
Gender, female	10	234	2.40 [0.93–6.16], (0.069)
Creatinine clearance: Ccr (mL/min)	58.9 ± 23.5	69.0 ± 25.3	0.98 [0.96–1.02], (0.096)
History of bleeding	1	78	0.46 [0.06–3.45], (0.449)
New users	8	346	1.28 [0.50–3.29], (0.604)
Change from warfarin	8	224	1.66 [0.65–4.27], (0.292)
C: Congestive heart failure	6	132	1.84 [0.67–4.99], (0.233)
H: Hypertension	9	417	0.56 [0.22–1.45], (0.231)
A: Aged 75 or older	13	281	3.16 [1.11–8.96], (0.031*)
D: Diabetes Mellitus	5	135	1.36 [0.47–3.90], (0.561)
S: Stroke/TIA (2 points)	10	175	3.24 [1.26–8.36], (0.015*)
CHADS2 score	2.94 ± 1.51	1.92 ± 1.34	1.69 [1.21–2.37], (0.002*)
With antiplatelet agents	2	127	0.55 [0.12–2.43], (0.426)

*P < 0.05

The incidence of thromboembolism was higher in patients with aged 75 years or older and stroke/TIA history. The factor of aPTT > 60 was not shown because some patients had incomplete data.

Among the factors shown in Table 2, the factors related to thromboembolic events were dose, age, A (aged 75 or older), S (stroke/TIA), and CHADS_2_ score. Dose, hypertension, aged 75 or older, and stroke/TIA history were selected as variables since the value of Akaike’s Information Criterion (AIC) was lower in this model. In these variables, dose and stroke/TIA history were selected by stepwise logistic regression method (Table 3).

**Table 3:** Factors that influence thromboembolic events by stepwise logistic regression method

Factor	Adjusted ODDS ratio	95% CI	*P*-value
Very low-dose (75 mg twice daily)	6.88	1.67–28.3	0.008*
H: Hypertension	2.25	0.83–6.12	0.110
A: Aged 75 or older	2.76	0.92–8.23	0.068
S: Stroke/TIA	2.97	1.11–7.94	0.030*

*P < 0.05

### Bleeding events

Major bleeding events occurred in 11 patients (Table 4) during the study period: 3 intracranial hemorrhages, 7 gastrointestinal bleeding, and 1 anemia case with no identified bleeding source. Only 1 event occurred in the 150 mg twice daily group (0.5%), and the other events were reported in patients receiving 110 mg twice daily (2.0%). Hemorrhagic complications (any bleeding including minor bleeding) were observed in 57 patients, and no significant difference was observed among each group. In the 75 mg twice daily group, 3 patients had minor bleeding. The incidence of hemorrhagic complications may be higher in patients who had lower CCr, were new users, changed to dabigatran from warfarin, were aged 75 years or older, had a stroke/TIA history, or received antiplatelet agents (Table 5). New users, aged 75 or older, stroke/TIA history, and “with antiplatelet agents” were selected as variables since the value of AIC was lower in this model. In these variables, new users and “with antiplatelet agents” were selected by stepwise logistic regression method (Table 6).

**Table 4:** Number of bleeding cases by dose of dabigatran

Classification of bleeding	Total (*n* = 701)	150 mg and 110 mg twice daily (*n* = 675)	75 mg twice daily (*n* = 26)	*P*-value
Major bleeding	11 (1.6%)	11 (1.6%)	0 (0.0%)	1.000
Any bleeding	57 (8.1%)	54 (8.0%)	3 (11.5%)	0.461

**Table 5:** Bleeding cases by dabigatran dose and each factor

Factor	Bleeding (+) (*n* = 57)	Bleeding (−) (*n* = 644)	ODDS ratio [95% CI], (*P*-value)
Very low-dose (75 mg twice daily)	3	23	1.50 [0.44–5.16], (0.520)
Age, years	74.0 ± 9.0	70.9 ± 11.0	1.03 [1.00–1.06], (0.036*)
Gender, female	19	225	1.07 [0.60–1.90], (0.808)
Creatinine clearance: Ccr (mL/min)	61.6 ± 29.4	69.4 ± 24.9	0.99 [0.97–1.01], (0.038*)
History of bleeding	9	70	1.53 [0.72–3.27], (0.264)
New users	19	335	0.46 [0.26–0.82], (0.007*)
Change from warfarin	26	206	1.81 [1.05–3.12], (0.034*)
C: Congestive heart failure	12	126	1.01 [0.51–1.96], (0.987)
H: Hypertension	38	388	0.76 [0.42–1.39], (0.376)
A: Aged 75 or older	33	261	1.78 [1.02–3.10], (0.043*)
D: Diabetes Mellitus	9	131	0.67 [0.32–1.39], (0.282)
S: Stroke/TIA (2 points)	24	161	2.00 [1.15–3.51], (0.015*)
CHADS2 score	2.44 ± 1.34	1.90 ± 1.34	1.33 [1.09–1.61], (0.005*)
With antiplatelet agents	17	112	2.02 [1.10–3.69], (0.022*)

*P < 0.056

**Table 6:** Factors that influence bleeding as side effect by stepwise logistic regression method

Factor	Adjusted ODDS ratio	95% CI	*P*-value
New users	0.51	0.27–0.91	0.022*
A: Aged 75 or older	1.55	0.87–2.79	0.136
S: Stroke/TIA	1.71	0.94–3.07	0.081
With antiplatelet agents	2.04	1.07–3.75	0.030*

*P < 0.05

## Discussion

The AF guidelines recommend the administration of anticoagulant drugs including dabigatran if the CHADS_2_ score is 1 or higher [9]. Although dabigatran can decrease the risk of a thrombus, there is a possibility of hemorrhagic side effects. Therefore, some patients may occasionally receive an excessively reduced dose of dabigatran, which is unapproved in Japan, to lower the risk of bleeding. It has been over 5 years since dabigatran was used clinically in Japan; thus, this analysis was performed and included patients using 75 mg twice daily.

Approximately 70% of the subjects in this study received 110 mg dabigatran twice daily. This tendency is similar to that reported in other Japanese studies [10, 11]. Patients receiving this reduced dose were older and had a lower CCr than patients receiving 150 mg twice daily; therefore, it was inferred that age and renal function were considered when selecting the dose of dabigatran. In the 75 mg twice daily group (26 patients), only 6 patients were newly started on anticoagulant therapy, 7 patients had the dose of dabigatran reduced, and 10 patients were switched to dabigatran from warfarin. The reason for the dose reduction to 75 mg twice daily varied from case to case; however, almost all of the patients on the very low-dose in this study were older than 70 years except for 3 patients, and some of them had aPTT prolongation, minor bleeding such as anemia or bloody stool, or renal dysfunction, or were on the combination of dabigatran and an antiplatelet drug or a P-glycoprotein inhibitor. One or more of these factors were considered to be involved in the dabigatran dose reduction. On the other hand, 7 other patients did not have any factors other than advanced age; therefore, it is possible that their doses were reduced at the discretion of their physician [11].

The CCr of a few patients was less than 30 mL/min. These patients must be prevented from dabigatran use; however, a small number of inappropriate uses have been reported in the “real-world” [10–12]. It was assumed that there were unavoidable reasons such as when renal function was around the boundary line of contraindicative criteria, or when warfarin could not be used because of its side effect. It might be related to the fact that DOACs are sometimes prescribed at a reduced dose [11, 12].

The incidence of thromboembolism was approximately 2% in the 150 and 110 mg twice daily groups in our study. In the subgroup analysis of Asian patients in the RE-LY trial, the incidence of thromboembolic events was 1.39% in the 150 mg twice daily group and 2.50% in the 110 mg twice daily group (CHADS_2_ score, 2.2 ± 1.1) [5]. In a real-world observational study of Japanese patients in Tokyo Women’s Medical University Hospital, the incidence was 0.6% (95% CI, 0.08–2.3%; CHADS_2_ score, 1.9 ± 1.5) [11]. It was inferred that the incidence of thromboembolic events depends on the study design and patient background, including the CHADS_2_ score [13]. On the other hand, 3 of 26 patients (11.5%) in the 75 mg twice daily dabigatran group had a thromboembolic event (femoral vein thrombosis, lacunar infarction, and TIA), and the incidence was higher than others. Moreover, lacunar infarction occurred in one of the 3 patients, who was 96-years-old and with a CCr of 30 mL/min. The other patients were 80 years old and their CCr was over 50 mL/min. This illustrates why the very elderly and those with poor renal function may receive doses lower than that recommended on the package insert. In AF patients undergoing coronary revascularization with warfarin anticoagulant therapy, the incidence of stroke was 6.9% with a time in therapeutic range (TTR) ≥ 65%; however, the incidence of stroke increased to 15.1% with TTR < 65%. Inadequate control of warfarin’s anticoagulant effect leads to inadequate stroke prevention and markedly higher cumulative 5-year incidence of stroke and mortality rates [14]. Optimization of anticoagulant dose is crucial for stroke prevention.

Overall, in our study, hemorrhagic side effects were observed in 57 patients (8.1%), among which 1 case (0.5%) in the 150 mg twice daily group and 10 cases (2.0%) in 110 mg twice daily group were major bleeding. In the 75 mg twice daily group, major bleeding was not observed, although it may be because the total number of cases was small. In the sub-analysis of Asian patients in the RE-LY trial, the rate of major bleeding was approximately 2.2% [5], and the total rate of serious hemorrhagic events in the post-marketing surveillance was 0.55% [10]. The incidence of hemorrhagic complications (any bleeding) may be higher in patients with lower CCr, aged 75 years or older, with stroke/TIA, or on antiplatelet agents (Table 5). Therapy change from warfarin to dabigatran was also found to be a risk factor for bleeding, perhaps because the patients who switched medication were poorly controlled with warfarin. In contrast, “New users” reduced the risk for bleeding. Major bleeding rates with dabigatran use were similar to those with warfarin use in real-world settings, and not in a randomized controlled trial [15].

Three subjects who had a thromboembolic event were excluded from this study owing to poor adherence. One patient discontinued treatment owing to an itching sensation. The other 2 patients only took dabigatran once a day. The half-life of dabigatran is shorter than that of warfarin, which is an advantage since the drug withdrawal period in case of surgery is shorter with dabigatran. However, poor adherence will attenuate the anticoagulant effect of dabigatran, and the risk of cardiogenic cerebral infarction will temporarily increase. A limitation of this study is that, owing to its retrospective nature, adequate adherence survey could not be performed.

## Conclusions

In this study, we analyzed the efficacy and safety of dabigatran including patients on a very low-dose of 75 mg twice daily. A limited number of patients were on this very low-dose, and none experienced a major bleed; however, 3 cases of thromboembolism occurred. In conclusion, the results suggest that sufficient anticoagulation efficacy may not be maintained when the dose of dabigatran is excessively reduced.

## Data Availability

The datasets used and analyzed during the current study are available from the corresponding author on reasonable request.

## Abbreviations

AF: Atrial fibrillation
AIC: Akaike’s Information Criterion
aPTT: Activated partial thromboplastin time
CCr: Creatinine clearance
CI: Confidence interval
DOAC: Direct oral anticoagulant
RE-LY: Randomized evaluation of long-term anticoagulant therapy trial
TIA: Transient ischemic attack
TTR: Time in therapeutic range
